# Chromosome evolution at the origin of the ancestral vertebrate genome

**DOI:** 10.1186/s13059-018-1559-1

**Published:** 2018-10-17

**Authors:** Christine Sacerdot, Alexandra Louis, Céline Bon, Camille Berthelot, Hugues Roest Crollius

**Affiliations:** 1 Institut de Biologie de l’Ecole Normale Supérieure (IBENS), Ecole Normale Supérieure, CNRS, INSERM, PSL Research University, 75005 Paris, France; 2 Present Address: Laboratoire Éco-Anthropologie et Ethnobiologie, UMR 7206 CNRS - Muséum National d’Histoire Naturelle, Université Paris Diderot, Sorbonne Paris Cité, F-75016 Paris, France

## Abstract

**Background:**

It has been proposed that more than 450 million years ago, two successive whole genome duplications took place in a marine chordate lineage before leading to the common ancestor of vertebrates. A precise reconstruction of these founding events would provide a framework to better understand the impact of these early whole genome duplications on extant vertebrates.

**Results:**

We reconstruct the evolution of chromosomes at the beginning of vertebrate evolution. We first compare 61 extant animal genomes to reconstruct the highly contiguous order of genes in a 326-million-year-old ancestral *Amniota* genome. In this genome, we establish a well-supported list of duplicated genes originating from the two whole genome duplications to identify tetrads of duplicated chromosomes. From this, we reconstruct a chronology in which a pre-vertebrate genome composed of 17 chromosomes duplicated to 34 chromosomes and was subject to seven chromosome fusions before duplicating again into 54 chromosomes. After the separation of the lineage of *Gnathostomata* (jawed vertebrates) from *Cyclostomata* (extant jawless fish), four more fusions took place to form the ancestral *Euteleostomi* (bony vertebrates) genome of 50 chromosomes.

**Conclusions:**

These results firmly establish the occurrence of two whole genome duplications in the lineage that precedes the ancestor of vertebrates, resolving in particular the ambiguity raised by the analysis of the lamprey genome. This work provides a foundation for studying the evolution of vertebrate chromosomes from the standpoint of a common ancestor and particularly the pattern of duplicate gene retention and loss that resulted in the gene composition of extant vertebrate genomes.

**Electronic supplementary material:**

The online version of this article (10.1186/s13059-018-1559-1) contains supplementary material, which is available to authorized users.

## Background

**Fig. 1:**
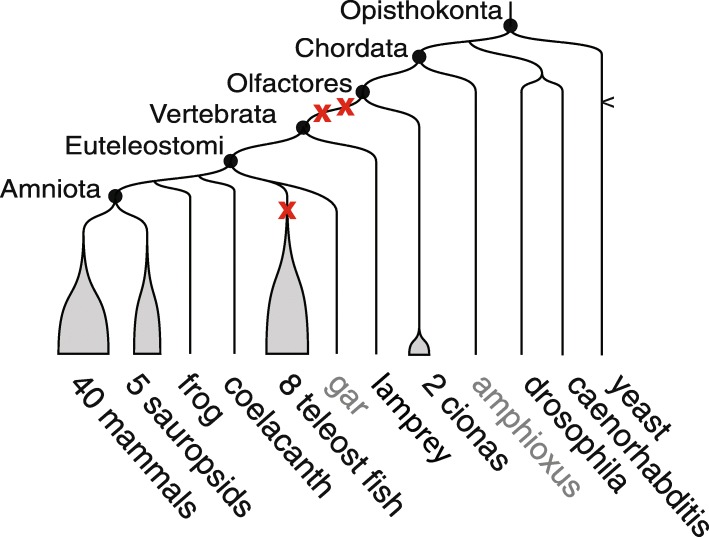
Schematic phylogenetic relationships between species used in this study. Species or groups of species shown in black at the end of branches are the 61 species included in Ensembl release 69, and were used to reconstruct the ancestral *Amniota* genome. Sauropsids include birds and reptiles. Species shown in grey were used at other stages in the analysis. Black circles materialize ancestral genomes relevant to this study. Red crosses indicate the relative positions of WGDs: two before the vertebrate radiation and one before the teleost fish radiation. Branches are not to scale

New gene copies largely appear by small-scale duplication during genome evolution [1], contributing to genetic innovation and phenotypic diversity [2]. In vertebrate evolution, whole genome duplications (WGDs) are rare, in contrast to plants where they appear to be more frequent [3]. More than 450 million years ago, an early vertebrate lineage was subject to two WGD in relatively rapid succession prior to its diversification into about 60,000 extant species (Fig. 1). Envisioned by Susumo Ohno since the early 1970s [4], these events known as the “1R-2R hypothesis” have since been firmly established by several genome-wide studies [5–7].

Approximately 35% of extant human genes still exist in duplicate copies (ohnologs) owing to the 1R-2R WGDs [8, 9], including the four clusters of HOX genes found in all tetrapods [10]. Ohnologs represent the vast majority of duplicated genes in the human genome [11]. They have been shown to be enriched in disease genes [8, 12–14] and to influence the frequency of structural variations in human populations [15]. The ancient 1R-2R genome duplications therefore still exert a strong influence on present-day genomes, warranting a better understanding of their early history. The first reconstruction of the evolution of the karyotypes before, during, and after the 1R-2R [6] left a number of questions unanswered. It could not reliably identify all ancestral vertebrate chromosomes nor could it determine if chromosome fusions or fissions took place between the two WGDs. This low resolution was due to the many chromosome rearrangements that have occurred since the vertebrate ancestor (*Vertebrata*), scrambling the ancestral gene organization in extant genomes, and the absence of a suitable outgroup species. In addition, a recent comparison between the lamprey genome and the chicken genome questioned the 1R-2R hypothesis and suggested that a single WGD and multiple segmental duplications could also explain the synteny patterns observed [16, 17] between these two species. Both the uncertainties of the first reconstruction of the pre-1R genome and this alternative to the 1R-2R hypothesis motivate a detailed analysis of the early evolution of vertebrate chromosomes.

The number of sequenced vertebrate genomes has recently greatly increased, allowing the reconstruction of ancestral genomes with much higher accuracy. After reconstructing the ancestral *Amniota* genome as a stepping stone to better understand the details of early vertebrate karyotype evolution, we find that the pre-1R vertebrate genome contained 17 chromosomes and that all vertebrates descend from a post-2R genome comprising 54 chromosomes. We show that the human genome still bears a strong imprint of the pre-1R genome, and provide resources to study the impact of the two WGDs in vertebrates from the perspective of a reference point shared by all descendent species.

## Results

### Identification of pairs of ohnologous genes in the ancestral *Amniota* genome

We inferred from Ensembl gene trees (version 69) that 19,786 genes existed in the ancestral *Amniota* genome, the ancestor of birds, reptiles, and mammals (326 million years). We used AGORA (Algorithm for Gene Order Reconstruction in Ancestors) [18] to order and orient these genes as they were in the *Amniota* genome (Additional file 1: Figure S1). This in silico reconstruction is composed of 470 segments, with 50% of the genes in segments larger than 253 genes (N50 length). We then selected the 56 chromosome-size segments larger than 50 genes as an initial set of Contiguous Ancestral Regions (CARs; mean CAR length 256 genes, 12,134 genes total) to identify duplicated regions.

Ohnologs resulting from the 1R-2R WGDs are key to identifying pairs of duplicated chromosome segments. We identified pairs of putative ohnologous genes directly in the reconstructed *Amniota* genome using gene trees to date their duplication while ensuring that each member of a pair belongs to a different CAR (see the “Methods” section), resulting in a “List A” containing 5616 ancestral *Amniota* ohnolog pairs. Two previous studies have also identified ohnologs from the two WGDs, in the human and other vertebrate genomes, using conserved synteny and sequence similarity. We used Ensembl gene trees to convert extant gene identifiers from these studies to their ancestral *Amniota* gene identifiers. The first list established by Makino and McLysaght [8] and hereafter called “List B” contains 4870 ancestral *Amniota* gene pairs. The second study by Singh et al. [9] established three levels of confidence (strict, intermediate, and relaxed) to define ohnologs. Following these criteria, we defined three additional lists containing respectively 2873 (List “C-strict”), 5253 (List “C-inter”), and 7806 (List “C-relax”) ancestral *Amniota* ohnolog pairs.

**Fig. 2:**
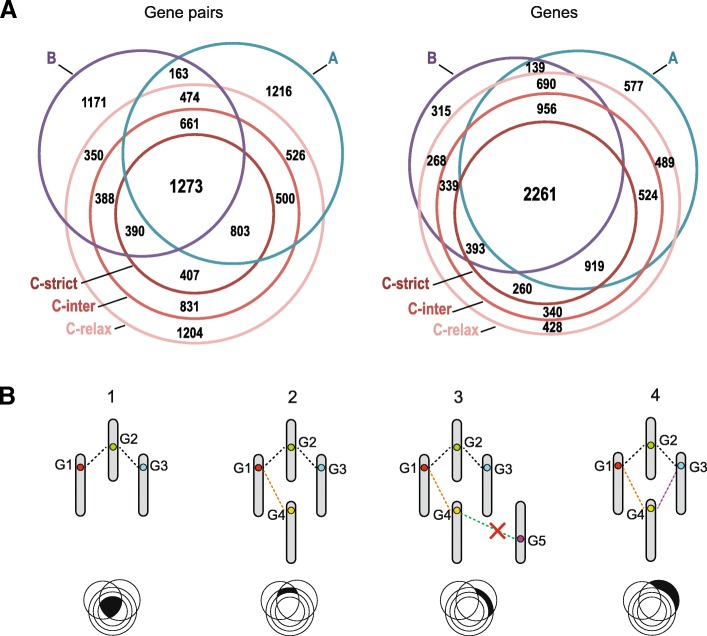
Identification of ohnolog pairs in the ancestral *Amniota* genome. (**a**) Comparison between five lists of ohnolog pairs in *Amniota*. Left: a Venn diagram of the sets of ohnolog pairs from five lists: list A (this study), list B [8] and the three lists C [9]. The numbers of pairs at the intersections of the lists are indicated. Right: a Venn diagram of the sets of ohnolog genes from the same lists as above. The overlap between the lists of ohnolog genes is higher than between the lists of pairs because the latter contain different pairs between the same genes. For example, two pairs G1-G2 and G1-G4 are in different lists (no overlap between lists) but gene G1 is common to both lists (1 gene overlap; see (**b**) for a graphical illustration). The surface of the circles and their intersection are roughly proportional to the number of genes pairs or genes of each list. (**b**) Schematic example of ohnolog pair selection. Step 1: from the initial list of 1273 gene pairs (black area in Venn diagram), 2 pairs involve 3 genes G1, G2, and G3, each on a different CAR. Step 2: pairs from a new sub-list are considered, a new gene pair G1-G4 is added to the network. Gene G4 is on a fourth CAR. Step 3: A new list is considered, a new pair is identified (G4-G5) but G5 is on a fifth CAR so pair G4-G5 is discarded. Step 4: a new list is considered, a pair G4-G3 supporting the network is identified

The sum of the A, B, and C-relax lists shows only 25% of genes in common (Fig. 2a), but we show below that the three lists nevertheless support the 1R-2R hypothesis. In this scenario, each original chromosome is duplicated in two then four copies; chromosomes thus form tetrads where each possesses three ohnologous counterparts. We tested whether pairs of CARs share more ohnologs than expected if they were distributed randomly using each of the five lists of ohnologs (proportionality test; see the “Methods” section). We show that, in all cases, CARs are ohnologous to three other CARS on average (Additional file 1: Figure S2). Despite their differences, all lists therefore support the 1R-2R hypothesis, justifying the construction of an improved consensus list of ancestral *Amniota* ohnolog pairs using all five lists. We started from the intersection of lists A, B, and C-strict as the most reliable subset (1273 pairs of ohnologs) and gradually extended it by adding pairs of genes from lower confidence subsets (pairs of genes intersecting fewer lists, or lists that include C-inter and C-relaxed; Fig. 2b). In this process, we ensured that the growing list remained compatible with the 1R-2R hypothesis: a pair could never be included if the two genes belong to two different phylogenetic trees (i.e., the two duplicated genes in a pair must descend from a common ancestral gene) and all the ohnologs within a phylogenetic tree, when arranged in pairs, cannot link more than four CARs (a tetrad; Fig. 2b and Additional file 1). This incremental process (Additional file 2: Table S5) resulted in a list of 8184 ohnologous genes, forming 7441 ohnolog pairs grouped into 2973 ohnolog families, each family in principle corresponding to one pre-1R gene (Additional file 3, 4, and 5 respectively for the list of ohnolog genes along with their human descendants, the list of ohnolog pairs and the list of ohnolog families).

This list of ohnolog pairs is of high quality for a number of reasons. First, it is based on the gene content and synteny in the reconstructed ancestral *Amniota* genome which is 326 million years closer to the 1R-2R events than extant genomes, and thus, signatures of 1R-2R events are read with greater accuracy. Second, this list abides by a 1R-2R-compatibility rule, i.e., no ohnologous gene family connects more than four CARs. Third, the ohnologous pairs are all phylogenetically consistent in that both genes in a pair always belong to the same Ensembl gene tree. Fourth, the two genes of a pair were allowed to be on the same CAR only if ≥ 90 genes separated them to avoid spurious inclusions of genes duplicated in tandem.

### Identification of post-2R duplicated CARs

Using our improved list of ohnolog pairs, we manually split, assembled, and grouped ancestral *Amniota* CARs in order to convert them to a configuration that is as close as possible to the post-2R karyotype. In the simplest scenario, post-2R CARs should readily form tetrads of four ohnologous CARs, each corresponding to one pre-1R chromosome. However, chromosome rearrangements between the 1R and 2R, between the 2R and *Amniota*, and incomplete or incorrect reconstruction of CARs all concur to disrupting this ideal pattern. We started with the 56 largest CARs and applied the proportion test to identify CARs sharing a significant number of ohnologous genes as described above (i.e., ohnologous CARs). We identified groups of at least three CARs all significantly ohnologous pairwise (*p* value < 5.10^−2^, Bonferroni adjusted). These were completed into tetrads (i.e., four CARs all significantly ohnologous to each other) by including smaller CARs and/or CARs at lower significance thresholds. We also merged CARs that showed evidence of belonging to the same *Amniota* chromosome (Table 1), because they were merged in alternative AGORA reconstructions using different sets of parameters, and/or they showed identical homologies to *Amniota* descendent genomes (human or chicken; Fig. 1). In addition, merged CARs had to be significantly ohnologous to at least one CAR in common in a triad and show no significant ohnology with each other. We also split CARs that showed, along their length, a disruption in their distribution of ohnologs and disruption of their homologies to chicken, human, spotted gar, or medaka chromosomes (Fig. 1). Finally, we confirmed merged CARs using homologies with outgroup species such as the spotted gar or the medaka (Fig. 1; see the “Methods” section).

**Table 1:**
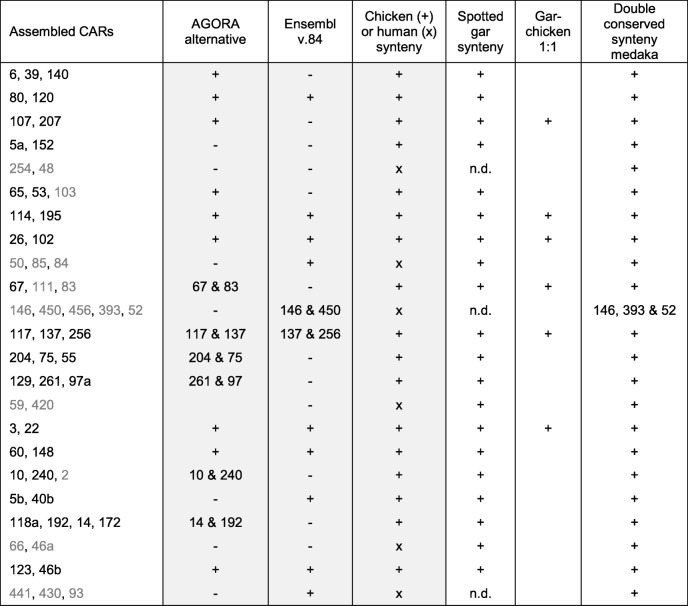
Assembly of *Amniota* CARs

Each line corresponds to the assembly of two to five CARs (column 1). “AGORA alternative” indicates that all CARs (+) or some CARs (CAR number shown) are merged as a single CAR in alternative AGORA reconstructions with different parameters. “Ensembl v.84” indicates that all CARS (+) or some CARs (CAR number shown) are merged in an AGORA reconstruction of the *Amniota* genome based on a more recent version of Ensembl (v. 84). “Chicken or human synteny” indicates that all assembled CARs are homologous to the same chicken (+) or human (x) chromosome. “Spotted gar synteny” indicates that assembled CARs are homologous to the same spotted GAR chromosome. Gar-chicken 1:1 indicates that assembled CARs are homologous to the same gar and chicken chromosomes. Double conserved synteny with medaka indicates that the assembled CARs are syntenic with the same pairs of chromosomes in the medaka genome. Greyed columns indicate that at least one of these conditions must be fulfilled for assembling CARs. Non-greyed columns are supporting evidence but dispensable conditions. A full description of the table can be found in Additional file 1

**Fig. 3:**
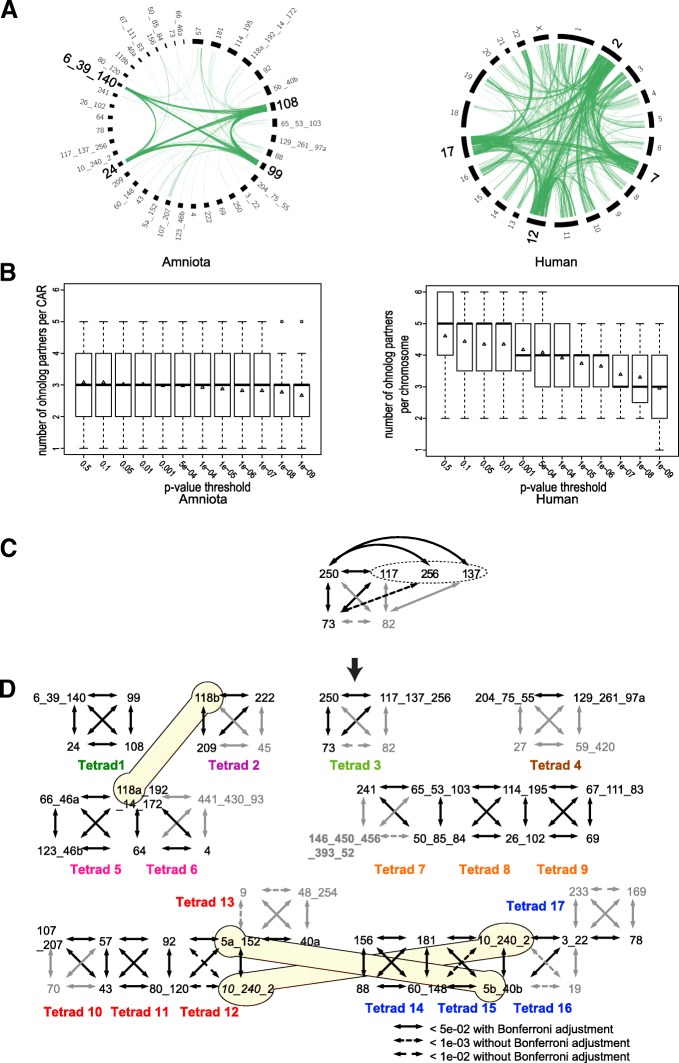
Organization of ancestral *Amniota* CARs in tetrads (**a**) Circos plot [45] showing the pairs of ohnologs involving each of the four chromosomes (*Homo sapiens*) or CARs (*Amniota*) of the tetrad carrying the Hox genes (Tetrad 1 in D). The pairs of ohnologs in the human genome were the descendants of those of *Amniota* (6121 pairs of human ohnologs vs. 7441 pairs of amniote ohnologs). The human Hox cluster tetrad is mainly composed of human chromosomes 2, 7, 12, and 17. The *Amniota* Hox cluster tetrad is composed of CARs 108, 24, 99, and 6_39_140. An ohnolog pair is represented (green lines) between two *Amniota* CARs or two human chromosomes if at least one of the two genes of the pair falls on a chromosome/CAR of the tetrad. The *Amniota Hox* CARs are involved in 634 pairs, while the human *Hox* chromosomes are involved in 2171 pairs of ohnologs. This figure shows that the reconstruction of *Amniota* ancestor displays a clearer picture of the 1R-2R than the human genome. (**b**) Ohnolog partners per CAR/chromosome in the *Amniota* (left) and human (right) genomes. Each boxplot shows the distribution of the number of CARs (*Amniota*) or chromosomes (Human) found to be ohnologous to a given CAR/chromosome by the proportionality test. The *x*-axis shows the Bonferroni adjusted *p* value thresholds used to select ohnologous chromosome/CARs. Triangles indicate the average number of partners. The *Amniota* genome shows a clear and stable distribution of three partners per CAR across a wide range of *p* values, as expected after two WGDs where chromosomes are grouped in tetrads. In contrast, the distribution in *Homo sapiens* shows that extremely low *p* value thresholds must be used to reach the expected average of three partners, justifying the fragmentation of the human genome as described in [6]. (**c**) Example of how a group of significantly ohnologous CARs was analyzed to form tetrad 3. Black double-headed arrows (*p* value < 5.10^−2^ after Bonferroni adjustment) represent the raw output of the proportion test, showing CARs with significant ohnology relationships. CARs 73, 117, and 250 form a triad of mutually ohnologous CARs. Dotted lines are additional ohnologous relationships that are supported without the Bonferroni adjustment. Numbers in black indicate CARs of at least 50 genes, while smaller CARs (< 50 genes) are in grey. Additional evidence (see text) was used to complete the tetrad. (**d**) Seventeen tetrads composed of 51 CARs. CARs are numbered arbitrarily and are joined by underscores in an arbitrary order when assembled. The letters “a” or “b” indicate that the CAR has been split in two segments (CARs 5 and 118) as part of the conversion to a post-2R karyotype (see text) and one CAR is present twice in two different tetrads (CAR 10_240_2) to facilitate the representation (pale yellow shapes)

The resolution of CARs in tetrads is much clearer after this conversion of *Amniota* CARs to a post-2R configuration, especially compared to human chromosomes (Fig. 3a). The proportionality test links each curated *Amniota* CAR on average to 3 other CARs almost independently of any *p* value threshold, whereas human chromosomes are much more sensitive to the *p* value threshold. Indeed in the human genome, the expected average of three partners per chromosome is reached only at a *p* value of 1.10^−09^ (Fig. 3b), a stringent threshold where 7 human chromosomes cannot be assigned to a tetrad. An example of construction of a tetrad and assembly of CARs is detailed in Fig. 3c. We identified three significantly ohnologous CARs grouped in a triad (CARs 73, 117, 250). Two additional CARs (256 and 137) show significant ohnology to CAR 250. The tetrad was completed with the addition of a smaller CAR (CAR 82), which was linked to four of the five initial CARs with significant but higher *p* values (Additional file 6). Then, of the five initial CARs, three fulfilled the conditions to be assembled in a single larger CAR (CARs 117, 137, 256): they were ohnologous to CARs in common but were not ohnologous to each other, and AGORA merged CARs 117/137 and CARs 137/256 together when using a more recent version of Ensembl (Version 84). Furthermore, the three CARs map to the same chicken and spotted gar chromosomes, strongly suggesting that they derive from the same chromosome of their common *Vertebrata* ancestor. Finally, all three assembled CARs, when mapped on the medaka genome (a teleost fish that went through an additional WGD [19]), are orthologous to the same two medaka chromosomes (13 and 14; Additional file 6).

We identified two post-2R chromosomal fusions that required a split of two *Amniota* CARs in two sub-CARs each (CARs 5 and 118), and we identified three probable assembly errors that required a split of three CARs (CARs 40, 46, and 97). We also performed 23 CAR assemblies (Table 1), ending with a final set of 51 CARs edited to more closely represent their post-2R configuration. The ohnology relationships between CARs based on significant *p* values of the proportion test connected the 51 CARs into 17 tetrads (Fig. 3d). The precise step-by-step procedure that we followed to split or assemble CARs and group them in tetrads is detailed in Additional file 1, including Additional file 1: Figure S7B to S14B.

### Chromosome evolution between the 1R and 2R whole genome duplications

**Fig. 4:**
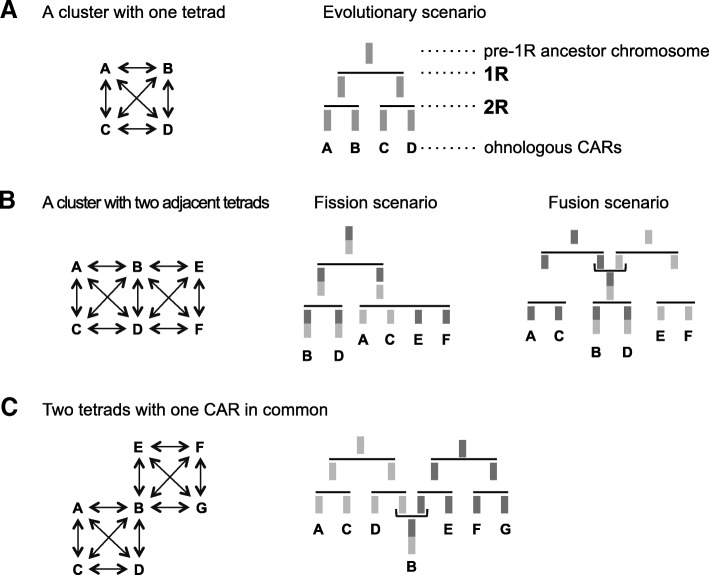
Evolutionary scenario models. (**a**) A single evolutionary scenario explains the formation of a single disjoint tetrad of ohnologous CARs. (**b**) Two equally possible evolutionary scenarios can explain how ohnologous CARs can form two adjacent tetrads: a fission or a fusion of chromosomes could have occurred between the 2 WGDs. In each case, the B and D chromosomes each possess two distinct parts (dark and light grey) homologous to distinct chromosome sets. B and D are therefore common to two tetrads. (**c**) A chromosome fusion after the two WGDs explains how two tetrads can be joined via a single CAR

The 17 tetrads composed of 51 *Amniota* CARs are not all disjoint: some share one or two CARs in common, reflecting chromosomal events between the 1R and the 2R, and after the 2R. We identified these events by first establishing theoretical scenarios corresponding to each configuration. A single disjoint tetrad implies a simple evolutionary scenario without any large chromosomal rearrangement between the two WGDs (Fig. 4a). Two adjacent tetrads, however, can be explained by one of two scenarios, each with the same degree of parsimony: a post-1R chromosome descending from a single pre-1R chromosome was broken (a fission), or two post-1R chromosomes, each descending from separate pre-1R chromosomes, were merged (a fusion; Fig. 4b). As previously noted [20, 21], a non-duplicated outgroup species would be helpful to discriminate between the two possible ancestral configurations. However, of the two nearest outgroups to vertebrates (Fig. 1), neither tunicates (e.g., species of the *Ciona* group) nor cephalochordates (e.g., the amphioxus *Branchiostoma floridae*) [7, 22] are suitable for this purpose. The former are too diverged to identify clear chromosome homologies, and the genome of *B. floridae* is too fragmented to be informative. To circumvent this problem, we used a previously published reconstruction of 17 Chordate Linkage Groups (CLG) (Additional file 1: Figure S15), which are groups of human genes descended from the same ancestral chordate chromosome [7]. This reconstructed proto-karyotype precedes the 1R-2R events by less than 50 million years and is located at a much shorter evolutionary distance to the *Vertebrata* ancestor than the extant amphioxus genome (Fig. 1).

Remarkably, each CLG was associated with one predominant CAR tetrad (Additional file 1: Figure S16). Consequently, all seven adjacent tetrads result from chromosome fusions between the 1R and 2R WGDs. Indeed, a chromosome fission would have split the gene content of a CLG over two different tetrads (Fig. 4b). Incidentally, we also found evidence for a chromosome fusion between the chordate ancestor and the 1R, because more than 75% of genes from CLGs 6 and 7 have their descendants in a single tetrad (tetrad 14; Additional file 1: Figure S16 and Additional file 7). Conversely, we could not confidently assign tetrad 13 to a CLG, likely because it is a small tetrad. We therefore conclude that the pre-1R karyotype comprised 17 chromosomes, duplicated into 34 chromosomes after the first WGD and followed by seven fusions. The resulting 27 chromosomes were duplicated in the second WGD leading to 54 *Vertebrata* chromosomes, at the origin of the approximately 60,000 extant species of vertebrates.

### Chromosome evolution after the 2R

This karyotype was followed by additional chromosome fusions at different stages after the 2R WGD. Four fusions followed the scenario described in Fig. 4c, where a single CAR joins two tetrads (CAR 5, CAR5a_152, CAR3_22, CAR 10_240_2; Fig. 3d). They can be dated to the period between the 2R WGD and the *Euteleostomi* ancestor because of their homologies to both descendent and outgroup genomes. For example, CAR_10_240_2 is homologous to a single chicken chromosome (GG4), a single human chromosome (chromosome X) and a single spotted gar chromosome (LG7), which is most parsimoniously consistent with a situation where this CAR was already a single chromosome in *Euteleostomi*, the common ancestor of these three species. A fifth fusion can be dated to the period between *Euteleostomi* and *Amniota*: CAR 118 is common to two tetrads but while it is homologous to a single chicken chromosome (GG1), a disruption of synteny in the spotted gar genome and a disruption in the DCS pattern in the medaka genome are consistent with a fusion in the lineage leading to *Amniota.*

**Fig. 5:**
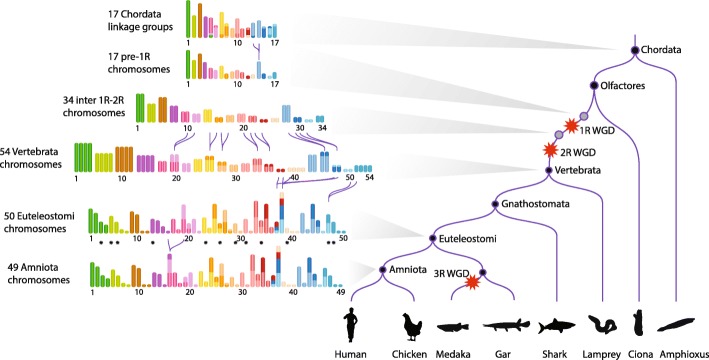
Reconstructed evolutionary history of karyotypes from *Chordata* to *Amniota*. On the right, a simplified species tree of the *Chordata* is shown, with WGD events depicted by red stars. The eight lineages represented from left to right are mammals, birds, teleost fish, holostocean fish (gar), cartilaginous fish, cyclostomes (lamprey, hagfish), tunicates (ciona), and cephalochordates (amphioxus). On the left, successive reconstructed karyotypes are shown, with one color for each of the 17 pre-1R chromosomes. The length of each pre-1R chromosome is proportional to its number of genes. For the 17 Chordate Linkage Groups (CLGs) of [7], the size of the colored segment is proportional to the number of genes that are found in the intersection of the CLG with a pre-1R chromosome, although segments corresponding to < 10% of the number of genes of the CLG were omitted for clarity (Additional file 1: Table S7). The karyotype between 1R and 2R was deduced from the pre-1R karyotype and the seven chromosome fusions are shown with purple curvy lines joining the fused chromosomes. The *Euteleostomi* karyotype was deduced from the *Vertebrata* karyotype after four chromosome fusions (Additional file 1). The lengths of the *Euteleostomi* chromosomes are proportional to the number of genes in the homologous *Amniota* CARs. Finally, the *Amniota* karyotype differs from that of *Euteleostomi* by only one chromosome fusion. The *Amniota* chromosomes were numbered from 1 to 49 (Additional file 1: Table S11 for correspondence with the CARs and number of genes). Black stars under 12 Euteleostomi chromosomes denote predicted ancestral micro-chromosomes

Accounting for these fusions, the 54 chromosomes in the post-2R *Vertebrata* led to a *Eueteleostomi* karyotype of 50 chromosomes (4 fusions) and to an *Amniota* karyotype of 49 chromosomes (1 fusion; Fig. 5 and Additional file 1). A dedicated Genomicus server [23] provides a graphical interface to compare and analyze the genomes presented in this work (Additional file 1: Figure S17; http://genomicus.biologie.ens.fr/genomicus-69.10/).

### Comparative genomics between the pre-1R genome and the human genome

**Fig. 6:**
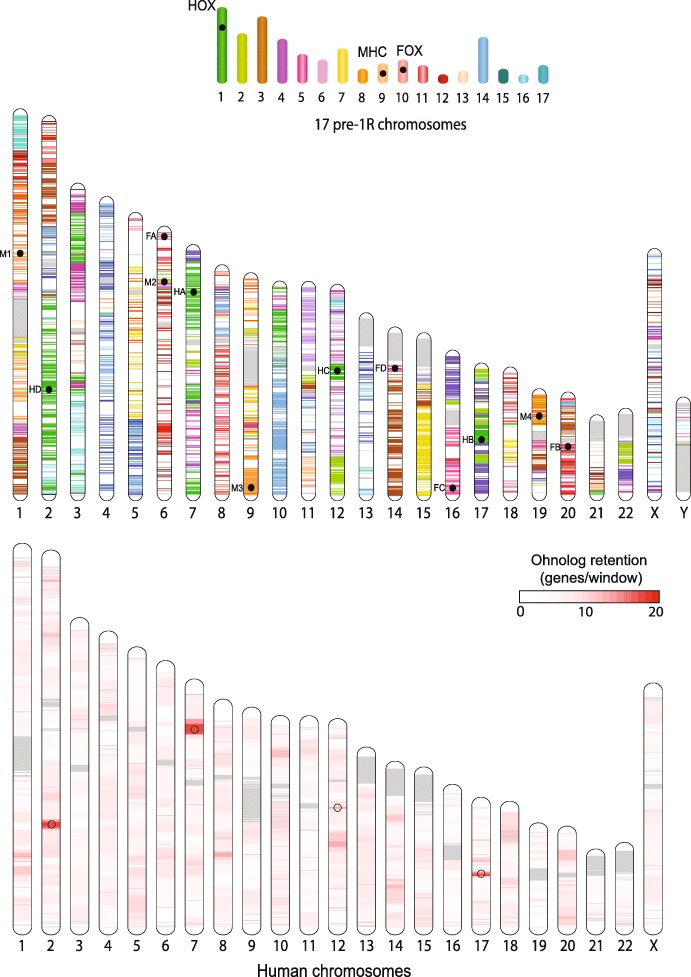
Comparison between the pre-1R karyotype (top) composed of 17 chromosomes and the human karyotype (middle). The 8282 known human descendent genes of pre-1R genes are drawn at their position in the human genome with the color of their pre-1R ancestral chromosome. The position of 12 extant clusters (4 HOX, 4 FOX and 4 MHC) descending from a single clusters in pre-1R chromosomes are indicated by a black circle and a 2-character identifier (M1, M2, M3, M4 for MHC clusters, F1, F2, F3, F4 for FOX clusters, HA, HB, HC, HD for HOX clusters). A second human karyotype (bottom) shows, in a white-to-red scale, the number of ohnologs in windows of 50 genes positioned every 10 genes. Open circles denote the position of HOX clusters. Human chromosomes are drawn to scale, in Mb. Pre-1R chromosomes are drawn as in Fig. 5, in proportion to the number of genes assigned to each. The order of genes in pre-1R chromosomes being unknown, the positions of the 3 pre-1R gene clusters within their chromosome are arbitrary

To enable comparisons between the pre-1R vertebrate ancestor genome reconstruction and extant species, we assigned genes to each of the 17 pre-1R chromosomes. Among *Amniota* genes, only ohnologs could be confidently assigned to pre-1R chromosomes, as non-ohnolog genes could have been acquired after the 1R WGD. To circumvent this problem, we applied a conservative procedure to assign 5052 of the 10,093 ancestral *Olfactores* genes to the 17 predicted pre-1R chromosomes (Additional file 8). The *Olfactores* ancestor is the common ancestor of vertebrates and tunicates and the closest ancestor upstream of the reconstructed pre-1R genome in Ensembl gene trees. This set of ancestral genes provides a direct connection to the human genome, through their 8378 human descendent genes. By identifying each of these human descendants by the color of its ancestral pre-1R chromosome (Fig. 6), we show that the structure of the 17 pre-1R chromosomes is still strikingly apparent in the human genome, with some chromosomes almost entirely composed of genes from a single pre-1R chromosome (e.g., chromosomes 14 and 15). We measured the degree of conservation of the post-1R-2R ohnolog content in windows of 50 genes positioned every 10 genes across human chromosomes. Three regions overlapping the Hox clusters A, B, and D stand out (and Hox C to a lesser degree; Fig. 6), in line with the known functional importance associated with the clustering of these ohnologs [24].

The four Hox clusters originate from pre-1R chromosome 1, and we examined in the same light other paralogous clusters that have been proposed to originate from a single pre-1R locus. The MHC region on human chromosome 6 contains a number of genes unrelated to immune functions but which possess ohnologs in 3 other loci on chromosomes 1, 9, and 19 [25]. All 4 regions descend from pre-1R chromosome 9. Similarly, the loci containing FOX gene clusters have been compared and found to share paralogs suggestive of en-bloc duplications early in vertebrate evolution [26]. We confirm here their unique origin on pre-1R chromosome 10. In contrast, no common origin was found for the different clusters of imprinted genes in the human genome (e.g., H19 locus on chromosome 11, IGF2R locus on chromosome 6, PON [1–3] locus on chromosome 7, UBE3A locus on chromosome 15) [27, 28], in line with their known progressive appearance later in therian mammals [29]. The evolutionary scenario of ancestral vertebrate chromosomes presented here is therefore consistent with our current view of the evolution of these important gene families.

Finally, we analyzed the frequency of Gene Ontology terms of the human descendants of the 1416 pairs (2 gene losses), 502 triplets (1 gene loss), and 172 quartets (no loss) of ancestral *Amniota* ohnologs and find a striking pattern: quartets are enriched in both neuronal development and neuronal function (synaptic transmission) and triplets are enriched in muscle development (especially heart) and in muscle function (contraction), while pairs (two losses) are enriched in protein maturation and transport between organelles (Additional file 9).

## Discussion

We analyzed 61 animal genomes to reconstruct the evolutionary history of genes and chromosomes in the lineage leading to the ancestor of vertebrates, which then diversified in more than 60,000 species in the course of the following 450 million years. In contrast to previous studies which analyzed extant genomes, we first carefully reconstructed the ancestral *Amniota* genome to identify the signature of chromosome duplications more clearly. Our rationale is that the reconstructed ancestral *Amniota* genome should be devoid of the noise caused by the numerous rearrangements that took place during the following 326 million years of evolution. Indeed, the benefit of this approach can be seen when comparing the distribution of ohnologs in the ancestral *Amniota* genome versus their descendants in the human genome, for example in the chromosomes carrying the *Hox* clusters (Fig. 3c). The expected four-way association between duplicated chromosomes is striking in the *Amniota* reconstruction but blurred in the human genome. Statistical confidence and power are thus higher (Fig. 3d), enabling a higher resolution of chromosome events than was previously possible. In turn, this higher signal-to-noise ratio enables a straightforward reconstruction strategy of pre-duplication genomes, without requirements for complex statistical steps or algorithmic developments (reviewed in [30, 31]).

Progress was made on understanding early vertebrate genome evolution when Nakatani et al. [6] reconstructed a vertebrate pre-duplication ancestral genome by segmenting the human genome into conserved vertebrate linkage groups. These were compared with other vertebrate genomes, especially with medaka, using tunicate and sea urchin genes to define ohnologs. Using this strategy, the authors inferred a karyotype of the pre-1R vertebrate ancestor, but could not resolve inter-WGD chromosome fusions or fissions, leading to the conclusion that between 10 and 13 pre-1R chromosomes existed. Here, using the ancestral *Amniota* genome instead of the human genome, we reconstruct 17 chromosomes for the pre-1R genome and find evidence for 7 fusions between the 2 WGDs but none for fissions. We therefore identify new chromosomes and find major differences between the two karyotypes (Additional file 1: Table S1). Our study provides an improved picture of ancestral vertebrate genome evolution in part because it is based on the reconstructed *Amniota* genome and also because it relied on information from more species and more recent genome annotations. For example the spotted gar genome [32] was more informative than the synteny with the medaka genome [19] because it lacks the numerous chromosome fusions that occurred before the teleost WGD [32]. Nakatani et al. also reconstructed an *Osteichthyes* post-2R genome corresponding here to the *Euteleostomi* ancestor, using a 2-of-3 rule relying on conservation between two of three genomes: the teleost pre-WGD, the chicken, and the vertebrate pre-1R genome [6]. Here however, our *Euteleostomi* ancestor is substantially different from this earlier study, since we describe an *Euteleostomi* genome of 50 chromosomes, not 31. This difference mainly comes from fusions that we believe were incorrectly inferred from chicken-teleost genome comparisons, which were confounded by high rates of chromosome fusions in the lineage leading to the ancestral teleost [32]. Indeed, given that the ancestral teleost fish possessed only 13 chromosome, the 50 chromosomes inferred here in the ancestral *Euteleostomi* suggest that the rate of chromosomes fusions in the lineage leading to teleosts must have been more intense than previously thought, in the order of 37 fusions in 100–150 million years. Similarly, the ancestral eutherian karyotype probably consisted of 23 pairs [33], suggesting a consistent pattern of karyotype reduction by chromosome fusion after the 1R-2R whole genome duplications. These fusions in the teleost and mammalian lineages involved both macro- and micro-chromosomes, probably explaining why these lineages do not possess micro-chromosomes any more.

**Fig. 7:**
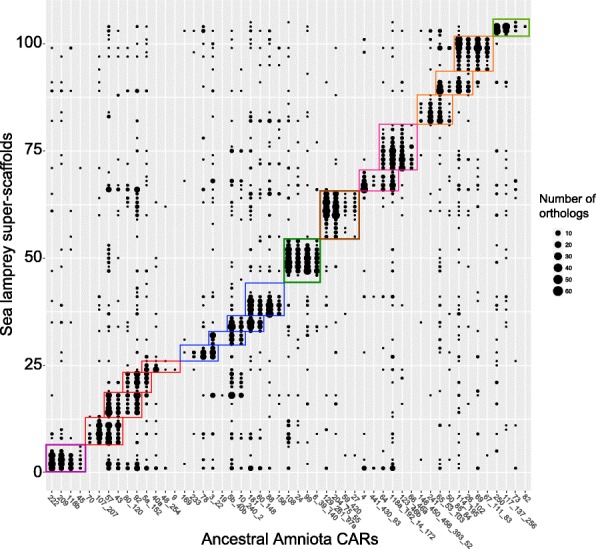
Comparison of ancestral *Amniota* CARs with super-scaffolds of the lamprey (*Petromyzon marinus*) germline genome assembly [17]. Along the *X*-axis, *Amniota* CARs are grouped in the 17 tetrads (colored boxes) as shown in Fig. 3d. The order of the lamprey scaffolds on the *Y*-axis was designed as to cluster them according to orthology pattern against *Amniota* CARs. The size of each black circle is proportional to the number of orthologs between a given *Amniota* CAR (X-axis) and the corresponding lamprey scaffold (*Y*-axis)

We addressed several questions using the improved picture of ancestral vertebrate chromosomes described here. First, it should be noted that neither the reconstruction of the *Amniota* genome nor the establishment of ohnolog pairs described here make any assumption about the existence of two successive WGDs early in vertebrate evolution. Indeed, the criteria used to select ohnologs only rely on duplication dates and on local synteny, leaving open the possibility that segmental duplications, a single WGD or two WGDs, have occurred during early vertebrate genome evolution. But the fact that *Amniota* CARs readily associate to form tetrads when we used ohnologous genes as links (Fig. 3b), is a striking confirmation of the 1R-2R hypothesis. This scenario is in fact largely agreed upon today, but the debate was recently re-opened [16] after a reconstruction of the pre-1R vertebrate genome using the lamprey (*Petromyzon marinus*) genome sequence [16, 17] and the chicken genome. Lamprey is a *Cyclostomata*, a sister group to *Gnathostomata* [34] to which Amniotes belong, and both groups share the pre-1R genome as common ancestor (Fig. 5). The lamprey genome is composed of almost 100 chromosomes, and its lineage separated from Gnathostomes soon after the last WGD. Redundant duplicate gene copies have therefore likely been lost largely independently in both lineages, leaving the possibility that a given Gnathostome (here chicken) chromosome would still display homologies to all lamprey chromosomes that derive from the same ancestral pre-1R chromosome [16]. Results of Smith et al. [16, 17] suggested that the ratio of ancestral pre-1R chromosomes to chicken chromosomes was mostly 1:2 and less frequently 1:4 or even 1:3, thus supporting a single WGD ancestral to both lineages combined with additional large numbers of segmental duplications, at least in the lamprey lineage. However, when replicating the above study with our reconstructed ancestral *Amniota* genome instead of chicken, a clear majority of 1:4 patterns appears (Fig. 7 and Additional file 1), hence supporting the occurrence of two successive WGDs. This further emphasizes the benefit of using ancestral genome reconstructions as intermediates when investigating such ancient evolutionary events (about 450 million years before present). In addition, the clear 1:4 pattern is most parsimoniously explained if the Gnathostomes and the lamprey lineages share the 1R-2R duplications in their common ancestral history, which places the divergence of the Gnathostomes from the lamprey lineage after the 1R-2R duplications.

The reconstructed *Amniota* genome is not complete, as the 49 chromosomes only contain 80% of the 15,854 ancestral *Amniota* genes assigned to CARs. We note that although all chromosome tetrads corresponding to pre-1R chromosomes are complete (i.e., are composed of 4 CARs), the 49 reconstructed *Amniota* chromosomes display large differences in gene numbers: the largest contains 862 genes (chromosome 37) and the smallest only 16 genes (chromosome 49). This could reflect either a more intense process of gene inactivation and loss on chromosomes with fewer genes, or a more intense rate of rearrangement on those chromosomes, leading to greater difficulties in reconstructing them. Small chromosomes with few genes do not follow any noticeable pattern in their distribution among tetrads, which might have indicated systematic biases in gene deletion during rediploidization. We further examined if chromosomes could be paired within a tetrad, as expected if gene loss following the first WGD left a distinct pattern on the two ohnologous chromosomes, that would have propagated to the two duplicates resulting from the second WGD. Here again, such a pattern is not noticeable, which may indicate that the two WGDs took place in rapid succession, as suggested before [35], leaving little time for gene deletions (diploidization) to leave their imprint. More high-quality genome sequences from extant amniotes are required to improve the reconstruction of their ancestral genome and identify more subtle patterns left by the 1R-2R. However, this current *Amniota* reconstruction and its ohnolog gene annotation already provide a solid foundation for new studies that should help resolve important questions, including complex phylogenetic histories [36].

An interesting question was raised during the analysis of the gar genome [32], when authors noticed a frequent 1:1 relationship between gar and chicken chromosomes, including micro-chromosomes. Micro-chromosomes are unusual because of their small sizes (usually below 20 Mb in chicken), their high GC content, high recombination rates, and high gene density. The gar-chicken comparison suggests that micro-chromosomes are ancestral features in *Euteleostomi*, which in turn raises the question of their origin through the 1R-2R duplications. Twelve gar and chicken micro-chromosomes are homologous and can parsimoniously be considered ancestral to *Euteleostomi* (Additional file 1: Table S2). They are distributed in 7 to 11 tetrads (some *Euteleostomi* micro-chromosomes originate from two tetrads), each of these tetrads containing both micro- and macro-chromosomes (Fig. 5). This leaves open the question of the timing of their formation: before the 1R, between the 1R and 2R, or immediately after the 2R.

The pre-2R karyotype and its evolution described here provide a framework to study the impact of the 1R-2R in extant vertebrate genomes, especially via the set of phylogenetically consistent ohnolog families that underlie the analysis. For example, previous studies have provided alternative hypotheses to explain a possible biased retention of ohnologs, including a requirement for stoichiometric balance of proteins in complexes [8, 37] or the preferential retention of genes involved in diseases owing to their dominant negative consequence on fitness if deleted from the genome [12, 38]. Ohnologs have also been shown to be enriched in developmental genes [39] and regulatory function [11]. A precise mechanism to explain biased ohnolog retention, if any, remains to be established. This may be because these studies are based on extant genes copies, and 500 million years of evolution have elapsed since the 1R-2R WGDs. Using the human descendants of ohnologous *Amniota* genes, we show that a striking pattern of biological functions are preferentially retained in ohnologous families that were subject to two gene losses, one gene loss or remained as a quartet of genes. Such distinctive and specific distribution of functions between the three types of ohnolog families raises the question of strong functional constraints governing early rates of ohnolog retention and loss. It is possible that these patterns reflect the functions and anatomical parts (central nervous system and muscles) most targeted by selection during early vertebrate evolution, when gene redundancy was maximum and provided a template for evolutionary innovations, leading to increased organismal complexity. This further illustrates the benefit of comparative genomics based on reconstructed ancestral genomes. 

## Conclusion

Biology is a *historical* science, but this historical dimension is often difficult to acknowledge because the genomic records required to document ancestral states are missing. In practical terms this lack of information hinders our ability to integrate conclusions made across different living organisms, and to draw all the benefits from comparative genomics. Here, the detailed reconstruction of the early history of the vertebrate genome is a step towards a better understanding of the founding events at the origin of living mammals, birds, reptiles, amphibians and fish. It provides a new perspective, that of a common reference, to study the evolution of these extant animals.

## Methods

### Phylogenetic gene trees

The complete set of 20,285 phylogenetic gene trees built by the Ensembl Compara pipeline (Ensembl version 69) [40] were downloaded using the Ensembl API. Duplication nodes were edited when their consistency score was below 0.3, as described in [41]. These phylogenetic gene trees were used in this study to (i) identify ancestral genes and orthologous gene relationships when reconstructing the *Amniota* ancestral genome, (ii) identify ohnologous gene relationships when constructing the “A” list of ohnologous gene pairs, and (iii) identify the ancestral and extant gene copies of genes from the original B and C lists of ohnologs and update the human-CLG relationships from reference [5] (all built using other Ensembl versions).

### Reconstruction of the *Amniota* ancestral genome

The ancestral *Amniota* genome was reconstructed using the AGORA (Algorithm for Gene Order Reconstruction in Ancestors) [18] (https://github.com/DyogenIBENS), which is routinely used to reconstruct ancestral gene order presented in Genomicus since Ensembl release 53. The *Amniota* reconstruction used here is available for download on the ftp site of the Genomicus webserver [23] (ftp://ftp.biologie.ens.fr/pub/dyogen/genomicus/69.10/ and [42]). AGORA takes as input the gene orders, gene orientations, and gene trees from 61 extant metazoan genomes available in release 69 of Ensembl (2012/11/15). The 61 species are listed in the Additional file 1: Table S4 and include 40 mammals, 3 birds, 1 reptile, 1 turtle, 1 amphibian, 1 coelacanth, 1 lamprey, and 8 teleost fish among vertebrates, and 2 Cionas, 1 fruit fly and 1 nematode. The corresponding species tree can be found at http://www.genomicus.biologie.ens.fr/genomicus-69.01/data/SpeciesTree.pdf

Briefly, AGORA is a graph-based parsimony method: it builds an adjacency graph where ancestral genes are vertices (nodes) and ancestral adjacencies are edges (links) weighted by the frequency of their conservation. This frequency is measured by comparing pairs of genomes descending independently from the *Amniota* ancestor (one *Mammalia* and one *Sauropsida*) or one descendant and one outgroup, and counting how many times a given ancestral gene adjacency is conserved between the two extant genomes being compared. The graph is then linearized along the edges of maximal weight to produce Contiguous Ancestral Regions (CARs). An AGORA reconstruction of the *Amniota* genome based on Ensembl version 84 (2016/03/15) was also used for comparison (Table 1).

### Identification of ohnolog gene pairs in the *Amniota* ancestor

Human ohnolog gene pairs from [8] based on Ensembl 52 data were assigned to their Ensembl version 69 gene trees using Ensembl gene IDs. Their *Amniota* ancestral genes were used to build list B of ohnolog gene pairs. Similarly, we downloaded three lists of ohnolog gene pairs from http://ohnologs.curie.fr/ described in [9], each list corresponding to a different degree of confidence level. A non-redundant list of *Amniota* ancestral genes was identified in Ensembl version 69 by sequentially identifying the *Amniota* ancestral genes of these ohnologs (data downloaded on October 27, 2014). We built list A by identifying ohnolog pairs directly in the *Amniota* reconstructed genome starting from ancestral genes that were duplicated between the *Chordata* and the *Euteleostomi* ancestors: these candidate pairs were considered ohnologs if another candidate pair from a different gene tree could be found on the same pair of CARs at a distance ≤ *N* genes (to account for massive duplicate loss after a WGD): the parameter *N* was made vary to optimize both sensitivity and specificity and fixed to 45 genes. A pair of ohnologs was allowed to occur between genes of the same CAR if they were located at a distance of 2*N* genes (90 genes, to account for possible rearrangements on the *Amniota* lineage), which added only 44 pairs. An integrated, high-quality list of ohnolog gene pairs was built from these five lists. Starting from their intersection consisting of 1273 pairs, we built disjoint graphs of ohnologs connected by ohnologous relationships. Replacing genes by the CARs they belong to, these graphs never involved more than four CARs of at least ten genes. To this core list, we sequentially added lists of pairs present in several or only one of the five initial lists, removing at each step the newly added pairs that would build ohnolog networks involving more than four CARs (Additional file 2: Table S5). The order in which these added lists were considered was established using the levels of confidence of the lists C, the number of lists where the pairs were found and the 1R-2R-compatibility criterion: a list was of better quality if it created fewer ohnolog networks of more than four CARs (per added pair) when added to the current validated list. Two properties of list A were maintained along the process: (1) the two ohnologs of a pair were allowed to be located on the same CAR only if they were ≥ 90 genes away from each other; (2) the two ohnologs of the pair had to belong to the same Ensembl gene tree.

### Identification of ohnolog CARs in the *Amniota* ancestor

Given the distribution of ohnologs on the *Amniota* CARs, a proportion test was performed (prop.test function in R) between each pair of CARs to estimate if the corresponding CARs shared more ohnolog pairs than expected by chance. Bonferroni adjusted *p* values of 0.05 or less were considered significant. However, *p* values obtained without the Bonferroni correction were also examined if CARs were included in a tetrad with a least one significant Bonferroni-adjusted *p* value.

### Attribution of ancestral *Olfactores* genes to the 17 pre-1R chromosomes

Phylogenetic gene trees from version 69 of Ensembl were analyzed to identify 10,093 nodes (genes) in the ancestral *Olfactores* genome, which were assigned to the 17 pre-1R tetrads according to the distribution of their descendent ancestral *Amniota* genes in the *Amniota* CARs used to build ohnologous tetrads. In the simple case of a single disjoint tetrad, all ancestral *Olfactores* genes with descendants exclusively in the 4 CARs of a tetrad were included in the corresponding pre-1R chromosome. Ancestral *Olfactores* genes with descendants in more than one tetrad were considered ambiguous and excluded. In the more complex cases of adjacent tetrads, the same principle was applied but when CARs belonged to two adjacent tetrads, only ohnolog genes were retained. In order to maximize the number of ancestral genes in the pre-1R chromosomes, we used all possible small CARs (≤ 50 genes) that could be assembled to the CARs of the tetrads, respecting all criteria of the 23 previous assemblies (Additional file 10).

### Human genes derived from ancestral chordate linkage groups (CLGs)

The coordinates of the 120 human chromosome segments from Tables S1 and S14 in reference [7] were updated from the hg18 to hg19 version of the human genome using the UCSC *liftOver* utility. Twenty-two segments were not converted by *liftOver* and were further fragmented in 100 sub-segments of equal size, which were mapped again to hg19 in order to recover as many genes as possible (Additional file 1: Figure S15).

### Gene Ontology (GO) analysis

The 2973 *Amniota* ohnolog families each contain from 2 to 11 ohnologs, depending on gene loss and duplications that occurred between the 1R and the ancestor of amniotes. To perform the GO analysis, we identified families containing a maximum of 4 ohnologs and where each ohnolog was located on a different CAR (1516 pairs, 502 triplets, 172 quartets). Ensembl gene trees were then used to identify human descendants of each ohnolog, if it exists. Lists of human genes were used on the Gorilla server [43], comparing each ohnolog list against the rest of the genome. After selecting GO terms enriched with an FDR < 10^−5^, terms were ranked by decreasing enrichment fold and the first 30 GO terms were analyzed and reported in Additional file 9.

## Additional files

Additional file 1: Additional methods, tables and figures, and a full description of all additional files. (DOCX 8000 kb)

Additional file 2: Describes the incremental construction of the list of ohnologs. (XLSX 18 kb)

Additional file 3: List of 8184 ancestral *Amniota* ohnolog gene names and their human descendent gene names (Ensembl gene ID). (TXT 336 kb)

Additional file 4: List of the 7441 pairs of ancestral *Amniota* ohnolog genes constructed in this work. (TXT 371 kb)

Additional file 5: List of the ohnolog families (ohnolog genes linked by pairs) from the list of ancestral *Amniota* ohnolog pairs in Additional file 4. (TXT 200 kb)

Additional file 6: Summarizes the evidence supporting tetrads. (XLSX 78 kb)

Additional file 7: Describes the comparison of the 17 tetrads to the 17 CLG of ref. [7]. (XLSX 83 kb)

Additional file 8: List of the composition of the 17 ancestral vertebrate chromosomes in terms of Olfactores ancestral genes (Ensembl gene ID). (TXT 459 kb)

Additional file 9: Results of the Gene Ontology enrichment analysis of human descendant of pairs, triplets, and quartets of ancestral *Amniota* ohnologs. (XLSX 57 kb)

Additional file 10: Summarizes the evidence supporting the addition of small CARs to tetrads. (XLSX 45 kb)
