# Supplementary material for: Chromosome evolution at the origin of the ancestral vertebrate genome
Source: Genome Biol. 2018 Oct 17;19:166. doi: 10.1186/s13059-018-1559-1 (PMC6193309; doi:10.1186/s13059-018-1559-1)
Supplement: Supplementary file 1 — Additional methods, tables and figures, and a full description of all additional files. (DOCX 8000 kb) [file 13059_2018_1559_MOESM1_ESM.docx]

**Chromosome evolution at the origin of the vertebrate genome**

Christine Sacerdot^1^, Alexandra Louis^1^, Céline Bon^1,2^, Hugues Roest Crollius^1^.

1. Institut de biologie de l’Ecole normale supérieure (IBENS), Ecole normale supérieure, CNRS, INSERM, PSL Research University, 75005 Paris, France.
2. New address: Laboratoire Éco-Anthropologie et Ethnobiologie, UMR 7206 CNRS - Muséum national d'Histoire naturelle - Université Paris Diderot, Sorbonne Paris Cité, F-75016 Paris, France.

**---**

**Additional file 1**

**----**

1. Building a list of ohnologous genes 2

2. Editing *Amniota* CARs and building tetrads 4

3. Using Chordate linkage groups (CLGs) to decipher chromosome events between the 2 WGDs 9

4. Post 2R fusions before and after the ancestor of *Euteleostomi* 10

5. Comparison with the lamprey genome 11

6. Description of tables and additional files 12

Table 1 *(main text)* 12

Additional file 2 13

Additional file 3 15

Additional file 4 15

Additional file 5 16

Additional file 6 16

Additional file 7 16

Additional file 8 17

Additional file 9 17

Additional file 10 17

Table S1 19

Table S2 20

Table S3 21

Table S4 22

7. Figs S1 to S16 23

## 1. Building a list of ohnologous genes

We started our analysis with 5 lists of ohnologous genes in the reconstructed Amniota genome. List “A” was established in the current study and consist in 5616 ancestral *Amniota* gene pairs (see methods). List “B” originated from a study by Makino & McLysaght ([Makino and McLysaght 2010](#_ENREF_2)) and consists in 4870 ancestral *Amniota* gene pairs converted from human gene identifiers to *Amniota* gene identifiers using Ensembl version 69 gene trees. List “C-strict”, “C-inter” and “C-relaxed” originated from a study by Singh et al. ([Singh et al. 2012](#_ENREF_5)) and consist in respectively 2873, 5253 and 7,806 *Amniota* gene pairs.

The five lists of ohnolog pairs overlap poorly (12% of the combined lists), but the corresponding lists of ohnolog genes show a better overlap (25%; Figure 2A). This is because the lists of ohnolog pairs group the same genes as different pairs, which can happen if after two rounds of WGD, each original gene is duplicated in two then (if no gene loss) into four copies, making six possible pairs, with each gene being involved in 3 pairs. Depending on the method to identify ohnologs and their homologies, not all remaining pairs are identified with the same sensitivity and specificity. However, we show that despite their differences all five lists support the 1R-2R hypothesis. Indeed, each original chromosome is duplicated in two then four copies (a tetrad). Thus, within a tetrad, each chromosome possesses three paralogous chromosomes. In the reconstructed ancestral *Amniota* karyotype, independently of the list used, a proportionality test (see methods) shows that CARs are, on average, linked by ohnolog pairs to three other CARS as expected (Fig. S1). We thus decided to construct an improved consensus list of ancestral *Amniota* ohnolog pairs using all five lists and without favoring one in particular, under a constraint of compatibility with the 1R-2R hypothesis.

A family of ohnologous ancestral *Amniota* genes is composed of genes that duplicated from a single pre-1R gene, and remain in the ancestral *Amniota* genomes after potential deletions and segmental duplications that took place between the *Vertebrata* and *Amniota* ancestors. In the simplest scenario, each gene in a family of ohnologs that was not subject to such deletions or additional duplications would be located on its own CAR, thus totaling four CARs per family. Some families may link fewer than four CARs, especially if some ohnologs were deleted post-2R. Some families may connect more than four CARs, for example if a post-2R gene duplication relocated a fifth copy on a fifth chromosome. However we reasoned that the main reason for families linking more than four CARs would be false positive ohnologs, i.e. genes that duplicated in another context than the 1R-2R and were incorrectly included in an ohnolog list. In support of this, we noted that the intersection of all five lists, i.e. ohnologs independently identified by three different methods and thus considered the least prone to errors, never connect more than four CARs within a family. This intersection comprises 1,273 pairs of ohnologs (Figure 2A). We show further that connecting a maximum of four CARs per family is a unique property of this intersecting list, because randomly drawing the same number of pairs from the complete union of lists (thus including potential false positives) always produces a list that has families connecting more than four CARs (Fig. S3). We call a family of ohnologs that does not connect more than four CARs a 1R-2R-compatible family. Starting from the set of 1,273 high-confidence 1R-2R-compatible pairs, we increased the list by adding pairs from sets of decreasing quality (Additional file 2), which either created a new family or complemented an existing family without connecting more than four CARs (Figure 2B).

The growing set thus remains 1R-2R-compatible. This incremental process (Additional file 2-tabA) resulted in a list of 8,184 ohnologous genes, linked in 7,441 ohnolog pairs grouped into 2,973 families, each family in principle corresponding to one pre-1R gene (Additional files 3, 4, 5 respectively for the list of ohnolog genes along with their human descendants, the list of ohnolog pairs and the list of ohnolog families).

The pairs are all phylogenetically consistent in that both genes in a pair always belong to the same Ensembl gene tree. This property was met by construction in list A, but not in list B (962 pairs of ohnologs, or 20%, belong to different trees) nor in the three lists C (5%, 9% and 15% of pairs in the strict, intermediate and relaxed lists C respectively belong to different trees). In the final list, the two genes of a pair were allowed to be on the same CAR only if ≥ 90 genes separated them to avoid spurious inclusions of genes duplicated in tandem. This criterion was also met when list C was used but was not met with the A and B lists (Additional file 2 – tab B).

## 2. Editing *Amniota* CARs and building tetrads

- 1. Splitting five CARs

Analysis of the dot matrix (Fig. S4) showed why some CARs were linked to a high number of other CARs based on the proportion test: CARs 5, 118, 40, 97, 46 each possess ohnologs with a distinct set of CARs, as expected if fusions of chromosomes took place since the *Vertebrata* ancestor. However, incorrect assembly of two disjoint ancestral *Amniota* chromosomes could give a similar result (Figs. S5 and S6). We therefore diagnosed each case in more detail and proceeded to split the CARs according to their ohnolog distribution.

- **CAR 5** (663 genes)

Visual inspection of the dot matrix of ohnolog genes in *Amniota* showed that it consisted in two segments that each displayed a specific set of ohnologous CARs. Each segment projects on the same chicken, gar and medaka chromosome and correspond to the same CAR in the AGORA reconstruction based on a more recent version (version 84) of Ensembl, suggesting that they are not an assembly error, but correspond to a chromosome fusion that took place after the 2R but before the split between Sarcopterygians and Actinopterygians (see section ‘**Post 2R fusions before and after *Euteleostomi*’** below). To account for the fact that each segment therefore was likely a different chromosome immediately after the 2R, CAR 5 was split in CAR 5a and CAR 5b at the precise position where the two distributions of ohnologs diverge.

- **CAR 118** (474 genes)

Visual inspection of the dot matrix of ohnolog genes in *Amniota* showed that it consisted of two segments that each displayed a specific set of ohnologous CARs: the first (left) segment of CAR 118 is ohnologous with CARs 4, 123, 64 and 46, while the second (right) segment is ohnologous with CARs 222 and 209. CAR 118 was split at the precise position where the two distributions of ohnologs diverge. CAR 118a and CAR 118b correspond to the same CAR in reconstructions using Ensembl version 84, project on the same chicken chromosome but on different gar and medaka chromosomes. This evidence indicate a correct assembly in *Amniota,* and that CAR 118 results from a post-2R fusions that occurred after *Euteleostomi*, on the lineage leading to *Amniota*. (see section ‘**Post 2R fusions before and after *Euteleostomi*’** below).

- CAR 40

CAR 40 was split between gene 120 and 121 in the AGORA reconstruction based on version 84 of Ensembl, the first 120 genes project on a different chicken chromosome and a different LG of the spotted gar than the segment beyond gene nr. 120, clearly suggesting an assembly error in the AGORA reconstruction based on version 69 of Ensembl, that was corrected in more recent versions.

- CAR 97 (92 genes)

CAR 97 was found to create pairwise ohnology relationships between five CARs (a pentad) instead of the expected tetrad. In addition, the proportion test showed that it was ohnologous to CAR 261, while evidence based on homologies to extant genomes (see below) showed that these two CARs should be assembled together. Careful examination of its projections on the chicken, human and spotted gar genomes, as well as the *Amniota* assembly in version 84, showed that synteny was consistently interrupted between the 72^nd^ and 73^rd^ gene (Fig. S5A): the segment of the first 72 genes project on chicken chromosome GG5, on spotted gar chromosome LG 7 and on human chromosome 15, while the short segment [73-92] has no projection on any chicken chromosome, projects on gar LG 2 and on human chromosome 19, leading us to split CAR 97 in two CARs 97a and 97b. After this split, the link between CAR 97a and CAR 261 was weaker but not completely suppressed (p-value = 5.1 10^-05^ without Bonferroni correction): examination of the two ohnolog pairs left between these CARs revealed a tandem duplication, which meant that only one actual pair linked them: this observation allowed us to consider them as non ohnologous CARs (Fig. S5B). The split of CAR 97 resolve the problem of the pentad and taken together, the evidence above suggests that the adjacency between the 72^nd^ and the 73^rd^ gene is an assembly error (Fig. S5).

- CAR 46 (77 genes)

CAR 46 was ohnologous to CAR 123 and yet had all properties to be assembled with it. However, all ohnologs of CAR 46 paired with an ohnolog on CAR 123 are located within the first 19 genes of CAR 46, which is the part of the CAR that projects on a different spotted gar LG and a different human chromosome from those of CAR 123, the latter having the same projections on these genomes as the downstream part of CAR 46 after gene 20 (Fig. S6). The evidence above suggests that the adjacency between the 19^th^ and the 20th gene is an assembly error. CAR 46 was therefore split between gene 19 and gene 20.

The split of 5 out of 56 CARs described above increased the total amount of CARs to 59 CARs larger than 50 genes.

2.2 Building tetrads

We applied the proportion test (methods) on the 59 CARs to build groups of CARs connected by ohnologous genes with a Bonferroni adjusted p-value threshold of 5.10^-2^. Ideally this graph should correspond to disjoint tetrads, i.e. independent sets of four ohnologous CARs. However, one CAR of the tetrad might be absent (not retained, or not reconstructed in the *Amniota* reconstruction, or not found because of biased massive loss of ohnologs) or evolutionary events such as fissions, fusions and rearrangements might have occurred between the two rounds of WGDs [[6](#_ENREF_6), [7](#_ENREF_7)], making the final pattern of links more complex than a simple tetrad. To account for this complexity, we conservatively initiated our search with triads (three CARS connected pairwise) instead of tetrads. Limiting the graph to the 59 CARs of at least 50 genes linked by ohnologs, we identified 8 disjoint groups of CARs, each containing at least one triad. With the same p-value threshold, we next added smaller CARs of at least 5 genes to these groups in order to complete triads into tetrads.

Most groups contained ohnolog links suggesting that some CARs should be assembled as part of the same ancestral *Amniota* chromosomes, i.e. they belong to the same group, share ohnolog links to common CARs and are not linked to each other. In addition, the assembly of these CARs is structurally supported in at least one of three additional ways (Table 1): (i) part of the same CAR in an AGORA reconstruction on the same version 69 of Ensembl but using alternative parameters when running the AGORA algorithm (ii) part of the same CAR in an AGORA reconstruction using a more recent version of Ensembl (version 84) (iii) common homologies to human and/or chicken chromosomes. These refinements led to 23 assemblies of two to five CARs within all but one tetrad (Table 1; Figure 3D, Additional Figs. S7B to S14B).

We describe below an example illustrating how CARs were assembled within tetrad 3. Five CARs larger than 50 genes, linked pairwise by significant p-values (p < 5.10-2, Bonferroni adjusted), formed tetrad 3 (Figure 3C). Three of the five CARS were linked in a triad (CARs 73, 117, 250), the minimal pattern that we required to form a group. The triad was turned into a tetrad with the addition of a CAR smaller than 50 genes (CAR 82, 9 genes), which was linked to four of the five initial CARs with significant p-values (Additional file 6). Then, of the five initial CARs, three fulfilled the conditions for being assembled into a single larger CAR (CARs 117, 137, 256): that is they all shared links to CARs in common and did not show any links to each other. Their assembly into a single CAR led to a perfect tetrad (Figure 3D). In addition, an alternative reconstruction with AGORA using different parameters placed CARs 117 and 137 together in a single scaffold, and a reconstruction of the Amniota genome using a more recent version of Ensembl (version 84) fused CARs 137 and 256 together. Furthermore, the three CARs map to the same chicken and gar (*Lepisosteus oculatus,* a non-teleostean fish) chromosomes, strongly suggesting that they derive from the same chromosome of their common *Vertebrata* ancestral genome. Finally all three assembled CARs, when mapped against the Medaka genome (a teleost fish that went through an additional WGD [[19](#_ENREF_19)]), show orthologous links to the same two Medaka chromosomes (13 and 14), as expected when a genome (here *Amniota*) is compared to a genome that went through a separate WGD (double conserved synteny pattern, DCS [[19-21](#_ENREF_19)]; Additional file 6). In combination, these three results strongly support the assembly of the three CARs.

All *Amniota* CAR assemblies performed during this step are supported by mapping on the same gar chromosomes, except for three sets of small CARs for which the gar chromosome could not be identified (n.d. in Table 1). All eight groups could be resolved into one to four tetrads (Figure 3B and Additional Figs. S7B to S14B).

## 3. Using Chordate linkage groups (CLGs) to decipher chromosome events between the 2 WGDs

The 17 CLGs of ([Putnam et al. 2008](#_ENREF_4)) are provided as sets of human genes (Fig. S15). On the other hand, ancestral *Amniota* genes from *Amniota* CARs grouped in tetrads (method) were replaced by their ancestral *Olfactores* genes to constitute a set of ancestral genes for each tetrad. To map CLGs (human genes) to the tetrads (*Amniota* tetrads mapped to *Olfactores* genes), the human gene content of each tetrad was deduced by taking all human descendants of these *Olfactores* genes. The 17 CLG sets of human genes were intersected with the 17 sets of human genes of the tetrads. The result of these 289 intersections is shown in Additional file 7, both in numbers of genes and in percentage of the CLG gene content. We observe that all 17 CLGs predominantly correspond to a single tetrad each, strongly indicating that no chromosome fission took place between *Chordata* and the pre-1R genome. Conversely, a single tetrad is associated to two CLGs (tetrad 14 with CLGs 6 & 7), showing that two CLG fragments translocated on the same pre-1R chromosome. Another tetrad (tetrad 13) has no corresponding CLG (Fig. S16). The latter tetrad contains few genes, which are mainly shared by CLGs 5 & 11, but represent a small part of their gene content.

## 4. Post 2R fusions before and after the ancestor of *Euteleostomi*

**- Pre-*Euteleostomi* events**

The two post-2R fusions that result from tetrads having only one CAR in common

The *Amniota* CARs 5a_152 and 3_22 each result from a post-2R fusion according to the scenario shown in Figure 4C, because they belong to two tetrads that have only one CAR in common. These fusions are independent from the assembly of the *Amniota* CARs 5a and 152 that both belong to tetrads #2c and #2d, and from the assembly of CARs 3 and 22 that both belong to tetrads 16 and 17

CAR 22 and the largest part of CAR 3 (325 genes, 220 orthologs, positions [1-309]) project on the same linkage group of the spotted gar (LG 14) and to the same chicken chromosome (GG9), indicating an ancestral *Euteleostomi* karyotype structure.

The post-2R fusion of CARs 5a and 5b

The split of CAR 5 into CARs 5a and 5b was achieved to convert the ancestral *Amniota* genome reconstruction into CARs that reflect the 4-way homology expected from 2 successive WGDs. However both parts of CAR 5 harbor the same pattern of projections on spotted gar LGs (LG17, LG3 and LG14) without any disruption of this pattern at the split point, indicating that the fusion occurred before *Euteleostomi*.

The post-2R fusion resulting in CAR 10_240_2

The post-2R fusion resulting in CAR 10_240_2 was inferred because this CAR belongs to two tetrads. The whole CAR projects on a single chicken chromosome (GG4), a single spotted gar LG (LG7) and on a single human chromosome (Hsa X), indicating that the fusion occurred before the *Euteleostomi* ancestor.

**- Post-*Euteleostomi* event**

The post-2R fusion of CARs 118a and 118b

In contrast to CARs 5a & 5b, the split point of CAR 118 matches exactly with a disruption of synteny with the spotted gar genome (LG8 + LG 1 for CAR 118a, and LG12 for CAR 118b) and a disruption in the pattern of Double Conserved Synteny with the *Medaka* genome (chromosomes 8, 1 and 19 for CAR 118a, and chromosome 26 for CAR 118b). Therefore the fusion of 118a & 118b occurred after the *Euteleostomi* ancestor, on the lineage leading to *Amniota*.

## 5. Comparison with the lamprey genome

([Smith et al. 2018](#_ENREF_7)) constructed a chromosome-scale assembly of the lamprey (*Petromyzon marinus*) germline genome. Using synteny conservation between the lamprey (representative of the Cyclostomata) and chicken genomes (representative of the *Gnathostomata*), they reconstructed the karyotype of their common Vertebrate ancestor. They reopened the 1R-2R debate and concluded to a more parsimonious scenario, where only one WGD and several segmental duplications occurred ([Smith and Keinath 2015](#_ENREF_6); [Smith et al. 2018](#_ENREF_7)). They found 13 ancestral pre-1R-2R chromosomes and based their conclusion of only one WGD on the fact that the ratio of ancestral to derived chromosomes was mostly equal to 1:2 (8/13 occurrences) rather than 1:3 or 1:4. We conducted a similar comparison but replacing the chicken genome by the ancestral *Amniota* genome reconstructed in our study, in the comparison with the ~100 chromosomes of the lamprey assembly. We found approximately the same conserved syntenic groups as in the study by ([Smith et al. 2018](#_ENREF_7)), but almost always forming clear tetrads (Figure 7, main text). Altogether, the comparison of the lamprey assembly with the ancestral *Amniota* genome rules out the 1R hypothesis and supports the 1R-2R hypothesis.

## 6. Description of tables and additional files

### Table 1 *(main text)*

This table describes the 23 assemblies of *Amniota* CARs that were performed within most tetrads. Each line corresponds to one assembly of two to five CARs. The CARs always share common ohnologous CARs (not shown, see Figure 3B, Figs. S7A to S14A, and Additional file 3) and are never linked to each other by the proportion test (see methods). Columns 2 to 4 indicate whether the structural conditions are fulfilled: (1) AGORA scaffolding, which was performed using 20 sets of parameters (data not shown, Louis et al. in preparation), was positive if the CARs were put together in at least one set of parameters; when the assembly involved more than two CARs, it happened that only a subset of these CARs be positive by AGORA scaffolding, in which case the CARs are indicated (for example: 67 & 83 in the assembly of 67, 111 and 83). (2) Some of the assemblies were found to be done in a more recent version of AGORA: version 84 (column 3); again when this occurred only for a subset of the CARs, the CARs involved are indicated. (3) Column 4 indicates whether the CARs map on the same chicken (*Gallus gallus*) chromosome. For some small CARs (with few genes) that had no (or too few) chicken descendants, the projection on the human genome was examined and the criterion was positive if they mapped on the same human chromosome. The last three columns show additional information that was not required for assembly but supports it: whether they project on the same spotted gar (*Lepisosteus oculatus*) linkage groups (LG), whether the CARs correspond to one of the twelve chromosomes that are in a 1:1 relationship between gar and chicken ([Braasch et al. 2016](#_ENREF_1)) and whether the CARs harbor the same pattern of double conserved synteny (DCS) with the *Medaka* genome. The CARs of less than 50 genes are indicated in grey.

### Additional file 2

(1A) Progression of the incremental process followed in this study to construct an improved consensus list of pairs of ohnologs in *Amniota* using five lists, four of them resulting from the remapping of ohnolog pairs identified in extant genomes in previous studies and the fifth one having been constructed in the ancestral *Amniota* genome in the present work (methods).

The progression from list n°1 to list n°14 was performed based on the following criteria:

First the B and C lists were filtered to remove pairs of ohnologs that belong to two different phylogenetic trees (from Ensembl V69), and the pairs of ohnolog located on the same *Amniota* CAR at a distance < 90 genes (Additional file 2 – tab B). The A list abides by these two properties by construction.

List n°1 is the intersection of all lists = the intersection of the A, B and C-strict lists. We next integrated additional sub-lists as described below (as schematized in Figure 2B), but removing, at each iteration, the pairs that added a 5^th^ CAR to the list of CARs already connected within a family of ohnologous genes.

- From list n°1 to n°2: intersection of three lists but relaxing the criterion of C-strict list to C-inter (= addition of the pairs of C-inter that are not in C-strict)

- From list n°2 to n°3 & n°4: intersection of two lists, but with the constraint that one of the two lists be C-strict: the two possible lists were ranked according to the number of ohnolog networks > 4 CARs that they created when added to list n°2: 0 for the list added to make list n°3 vs. 2 for n°4

- From list n°4 to n°5: we added the pairs that are in three lists but with the lowest criterion of C lists: C-relax (the pairs that are not in C-inter)

- From list n°5 to n°6 & 7: we relaxed the criterion of being in three lists to that of being in two lists, but with the constraint that one of the two lists be C-inter: again the two possibilities were ranked according to the number of ohnolog networks that they created when added to list n°5: 1 for the list added to make list n°6 vs. 3 for n°7

- In the next step, four lists could be added to list n°7. Their pairs either have the property of being in two lists or they fulfill the strict criterion of C; without knowing a priori which one was best, they were added using the criterion of the number of ohnolog networks > 4 CARs per added pair (to normalize for the differences in size) that they created when added to list n°7: 1 (0,6%), 4 (1,4%), 9 (1,7%) and 7 (2,8%) respectively for the lists added to make lists n°8, 9, 10 and 11.

- Last step: addition of the pairs that were specific to one list: A or B or C-inter (only the pairs specific to C-relax were not considered); this last set of three lists was ordered similarly as above: 36 (3%) unwanted networks were created by adding the A specific pairs to list n°11, while 25 (4,9%) and 31 (7,7%) such networks were created by adding the C-inter and A specific pairs respectively, to lists n°11.

The 14 steps are illustrated below the table: in black, the number of pairs for each surface with (in brackets) the number of pairs after filtering out the pairs of genes located on the same CAR at a distance < 90 genes or arising from different trees; in square brackets, the order of addition of the list, with the same color for lists in the same pool.

(1B) Table of the number of ohnolog pairs in each list, with the number of pairs where the ohnologs are on the same CAR (column 3), the number of cases where they are closer than 90 genes apart, in number of pairs and in % of the pairs of the list (columns 4 & 5), the number of cases where the ohnologs belong to different trees, in number of pairs and in % of the pairs of the list (columns 6 & 7).

### Additional file 3

List of 8,184 ancestral *Amniota* ohnolog genes (from the unbiased list of pairs, this work) with their human descendants in Ensembl v69, as a tabulated text file:

field 1 : ancestral *Amniota* gene
field 2 : human descendant gene(s) separated by a space.

### Additional file 4

List of 7441 pairs of ancestral *Amniota* ohnolog genes constructed in this work.

### Additional file 5

Ohnolog families (ohnolog genes linked by pairs) from the list of ancestral *Amniota* ohnolog pairs in additional file 4, with one family per line.

### Additional file 6

This table summarizes the main data for each group of CARs, with one tab per group of tetrads. In each case, a table indicates which CARs are in the group, their number of genes, to which CARs they are associated by AGORA, the name of the CAR where they map in version 84 of AGORA, on which chromosome(s) of chicken, human and spotted gar they map, and their pattern of DCS with the *medaka* genome. Below this table, the tetrads that form at specific p-value thresholds and all p-values pairwise above a given threshold are indicated in two versions of the ancestral *Amniota* genome: without conversion (raw output of AGORA) and after the 23 assemblies of CARs (described in Table 1). The colors highlight the common features of CARs that were assembled. The CARs of less than 50 genes are either in grey or in a color to underline assemblies.

### Additional file 7

Comparison of the 17 tetrads to the 17 CLG of ([Putnam et al. 2008](#_ENREF_4))

The first table of the first tab (“Gene numbers”) indicates the number of genes that each tetrad contains: the number of ancestral *Olfactores* genes, the number of human genes that descend from them and how many genes among them are found in a CLG. The second table of the same tab indicates the number of human genes that each CLG contains and how many of them are in a tetrad. Note that each CLG is named according to the original number of ([Putnam et al. 2008](#_ENREF_4)) (from 1 to 17).

Tab 2 (“CLG_tetrad_intersection_gene_nb”) shows the intersections of all tetrads with all CLGs in terms of number of genes, and tab 3 (“CLG_tetrad_intersection_%”) shows the same intersections in terms of the % of the number of genes in the CLG.

### Additional file 8

Reconstructed pre-1R genome as a tabulated text file:

Field 1: chromosome number

Field 2: ancestral *Olfactores* gene

Field 3: *Amniota* descendant genes separated by a space

Field 4: human descendant genes separated by a space.

The order of the genes in the chromosomes is arbitrary.

### Additional file 9

Gene Ontology enrichment analysis of human descendant of pairs, triplets and quartets of ancestral *Amniota* ohnologs.

### Additional file 10

All small CARs (< 50 genes) that were added to the tetrads in order to incorporate the maximum of ancestral *Olfactores* genes in the pre-1R genome are shown with the color of their group (column 1). Column 2 indicates to which CAR the small CAR (column 1) was assembled. Otherwise this table is comparable to table 1 but for the small CARs that were not necessary to identify the tetrads, but were used to assign genes to these tetrads. All these assemblies were confirmed also by ohnology links (data not shown).

| This work (tetrads) | This work (chromosomes) | Nakatani et al., 2007 |
| --- | --- | --- |
| 1 (*Hox*) | 1 | E |
| 3 | 2 | H |
| 4 | 3 | G |
| 2 | 4 | I |
| 5 | 5 | D |
| 6 | 6 |  |
| 7 | 7 | A |
| 8 | 8 |  |
| 9 | 9 |  |
| 10 | 10 | B |
| 11 | 11 |  |
| 12 | 12 | - |
| 13 | 13 | J |
| 14 | 14 | C |
| 15 | 15 | - |
| 16 | 16 | F |
| 17 | 17 |  |

### Table S1

Correspondence between the reconstruction of the pre-1R karyotype described in this study and the previous reconstructions from ([Nakatani et al. 2007](#_ENREF_3)).

| gar LG | chicken chromosome | *Amniota* CARs, v.69 |
| --- | --- | --- |
|  |  |  |
| LG14 | GG9 | 3, 22 |
| LG23 | GG11 | 107, 207 |
| LG13 | GG14 | 222 |
| LG20 | GG15 | 250 |
| LG21 | GG17 | 241 |
| LG22 | GG19 | 117, 137, 256 |
| LG18 | GG20 | 43 |
| LG25 | GG21 | 78 |
| LG26 | GG24 | 40a |
| LG24 | GG25 | 67, 111, 83 |
| LG15 | GG27 | 24 |
| LG19 | GG28 | 26, 102 |

### Table S2

Twelve spotted gar and chicken micro-chromosomes that are in a 1:1 relationship ([Braasch et al. 2016](#_ENREF_1)), along with the corresponding homologous *Amniota* CARs.

| *Amniota* chromosome | *Amniota* CAR | number of genes in *Amniota* CAR | added small CARs* | total number of genes including small CARs | |
| --- | --- | --- | --- | --- | --- |
| 1 | 108 | 510 | 105, 12 | 559 | |
| 2 | 99 | 406 | 151, 235 | 479 | |
| 3 | 24 | 253 |  | 253 | |
| 4 | 6_39_140 | 190 |  | 190 | |
| 5 | 250 | 355 |  | 355 | |
| 6 | 117_137_256 | 246 | 34, 44 | 314 | |
| 7 | 73 | 64 | 178 | 78 | |
| 8 | 82 | 9 | 15, 47, 106, 251 | 45 | |
| 9 | 129_261_97a | 434 | 138 | 482 | |
| 10 | 204_75_55 | 403 | 35, 163, 174 | 456 | |
| 11 | 59_420 | 38 | 32, 36, 131, 414 | 105 | |
| 12 | 27 | 38 | 104, 463, 74, 97b | 95 | |
| 13 | 222 | 308 | 23, 226, 260 | 355 | |
| 14 | 209 | 259 | 144, 175 | 299 | |
| 15 | 45 | 29 |  | 29 | |
| 16 | 192_14_172_118 | 731 |  | 731 | |
| 17 | 123_46b | 301 | 30 | 329 | |
| 18 | 66_46a | 60 |  | 60 | |
| 19 | 64 | 208 | 154, 196, 252 | 244 | |
| 20 | 4 | 304 |  | 304 | |
| 21 | 441_430_93 | 25 | 86 | 43 | |
| 22 | 69 | 343 | 187 | 352 | |
| 23 | 67_111_83 | 120 | 225, 257 | 141 | |
| 24 | 114_195 | 597 | 29, 72 | 642 | |
| 25 | 26_102 | 203 | 13, 109, 199 | 270 | |
| 26 | 65_53_103 | 490 |  | 490 | |
| 27 | 50_85_84 | 85 | 33, 71, 232 | 142 | |
| 28 | 241 | 201 | 37, 51, 56 | 258 | |
| 29 | 146_450_456_393_52 | 36 |  | 36 | |
| 30 | 107_207 | 287 |  | 287 | |
| 31 | 70 | 32 | 17, 62 | 68 | |
| 32 | 57 | 663 |  | 663 | |
| 33 | 43 | 274 | 135, 150, 234 | 323 | |
| 34 | 92 | 594 |  | 594 | |
| 35 | 80_120 | 189 | 126, 38 | 229 | |
| 36 | 10_240_2 | 251 | 76 | 283 | |
| 37** | 152_5_40b | 862 |  | **862** | |
| 38 | 40a | 120 | 54 | 159 | |
| 39 | 48_254 | 27 |  | 27 | |
| 40 | 9 | 6 | 1, 21 | 32 | |
| 41 | 88 | 417 | 202, 205 | 471 | |
| 42 | 156 | 101 | 79, 122 | 137 | |
| 43 | 181 | 603 | 98, 219, 220, 221 | 690 | |
| 44 | 60_148 | 272 | 136, 176 | 326 | |
| 45 | 19 | 9 | 8 | 23 | |
| 46 | 3_22 | 398 | 182 | 423 | |
| 47 | 78 | 238 |  | 238 | |
| 48 | 233 | 36 | 245 | 57 | |
| 49** | 169 | 16 |  | **16** | |
|  |  |  |  |  | |
| * CARs < 50 genes of the same group that could be assembled with the CAR of interest and were used to assign ancestral *Olfactores* genes to the appropriate tetrad (pre-1­­­­­R chromosome) | | | | |  |
|  |  |  |  |  |  |
| **The size of the largest and smallest chromosomes are indicated in bold | | | |  |  |

### Table S3

The 49 *Amniota* chromosomes in the order they are represented in figure 5 are listed with their corresponding CAR (column 2) and the number of genes of this CAR (column 3). Column 4 indicates the additional small CARs (< 50 genes) that were added to the CAR of column 2 in order to maximize the number of genes assigned to the pre-1R chromosomes (Additional file 4) and column 5 gives the total number of genes of the chromosomes after addition of these small CARs.

| **Species name** | **Common name** | Table S4 List of the 61 species whose protein-coding gene content and gene order were compared to reconstruct the ancestral *Amniota* genome. All the data was downloaded from the Ensembl version 69 website via the BioMart facility. The Ensembl gene IDs, gene positions and phylogenetic trees are available from the Genomicus website dedicated to the present study at the following address:  **http://www.genomicus.biologie.ens.fr/genomicus-69.10/** |
| --- | --- | --- |
| Ailuropoda melanoleuca | Panda |  |
| Anolis carolinensis | Anole lizard |  |
| Bos taurus | Cow |  |
| Caenorhabditis elegans | Caenorhabditis elegans |  |
| Callithrix jacchus | Marmoset |  |
| Canis familiaris | Dog |  |
| Cavia porcellus | Guinea pig |  |
| Choloepus hoffmanni | Sloth |  |
| Ciona intestinalis | Ciona intestinalis |  |
| Ciona savignyi | Ciona savignyi |  |
| Danio rerio | Zebrafish |  |
| Dasypus novemcinctus | Armadillo |  |
| Dipodomys ordii | Kangaroo rat |  |
| Drosophila melanogaster | Fruitfly |  |
| Echinops telfairi | Lesser hedgehog tenrec |  |
| Equus caballus | Horse |  |
| Erinaceus europaeus | Hedgehog |  |
| Felis catus | Cat |  |
| Gadus morhua | Cod |  |
| Gallus gallus | Chicken |  |
| Gasterosteus aculeatus | Stickleback |  |
| Gorilla gorilla | Gorilla |  |
| Homo sapiens | Human |  |
| Ictidomys tridecemlineatus | Squirrel |  |
| Latimeria chalumnae | Coelacanth |  |
| Loxodonta africana | Elephant |  |
| Macaca mulatta | Macaque |  |
| Macropus eugenii | Wallaby |  |
| Meleagris gallopavo | Turkey |  |
| Microcebus murinus | Mouse lemur |  |
| Monodelphis domestica | Apossum |  |
| Mus musculus | Mouse |  |
| Mustela putorius furo | Ferret |  |
| Myotis lucifugus | Microbat |  |
| Nomascus leucogenys | Gibbon |  |
| Ochotona princeps | Pika |  |
| Oreochromis niloticus | Tilapia |  |
| Ornithorhynchus anatinus | Platypus |  |
| Oryctolagus cuniculus | Rabbit |  |
| Oryzias latipes | Medaka |  |
| Otolemur garnettii | Bushbaby |  |
| Pan troglodytes | Chimpanzee |  |
| Pelodiscus sinensis | Chinese softshell turtle |  |
| Petromyzon marinus | Lamprey |  |
| Pongo abelii | Orangutan |  |
| Procavia capensis | Hyrax |  |
| Pteropus vampyrus | Megabat |  |
| Rattus norvegicus | Rat |  |
| Saccharomyces cerevisiae | Yeast |  |
| Sarcophilus harrisii | Tasmanian devil |  |
| Sorex araneus | Shrew |  |
| Sus scrofa | Pig |  |
| Taeniopygia guttata | Zebrafinch |  |
| Takifugu rubripes | Fugu |  |
| Tarsius syrichta | Tarsier |  |
| Tetraodon nigroviridis | Tetraodon |  |
| Tupaia belangeri | Tree schrew |  |
| Tursiops truncatus | Doslphin |  |
| Vicugna pacos | Alpaca |  |
| Xenopus tropicalis | Xenopus |  |
| Xiphophorus maculatus | Platyfish |  |

## 7. Figs S1 to S16

**Fig S1.** Strategy to reconstruct the pre-1R karyotype and deduce ancestral vertebrate chromosome evolution. Indicated at the top (green) are the two main data input to the strategy: lists of ohnologous genes and annotated genes from 61 animal genomes. Key analysis steps are depicted on orange backgrounds. Steps with purple background indicate the main result of the analysis shown in figure 5.

******A** **B**

**C** **D**

**E** **F**

**Fig S2.** Boxplots of the number of ohnologous partner CARs per CAR in the reconstructed *Amniota* genome (with the non-curated karyotype of the 56 CARs ≥ 50 genes), using six lists of ohnolog pairs: (A) the A list, (B) the B list, (C) the C-inter list, (D) the C-strict list, (E) the C-relax list, and (F) the final list of 7,741 genes used for the rest of the study. Triangles indicate the average number of partners

**Fig S3.** The 1R-2R-compatibility of the intersection of all lists of ohnolog pairs containing 1,273 pairs is a unique property that could not happen by chance. The figure shows that 1000 random draws (without replacement) of the same number of pairs (1,273) out of the 10,356 pairs present in the *union* of all lists results in a distribution where all draws generate lists with more than four 1R-2R-incompatible ohnolog families (= families of connected ohnologs that are altogether located on > 4 CARs) and where 98% of the random lists contain 6 to 16 such networks, with 59% of them having 9 to 12 such networks.

**Fig S4.** Dot-plot of the distribution of ohnolog genes along *Amniota* CARs (self-comparison)The ohnologous genes are those of the final list of pairs (8,184 ohnologous genes in 7,741 pairs). The *Amniota* CARs are sorted by decreasing size along both axes. The red vertical lines show the three largest CARs (5, 118, 40) that were split in two segments that each possesses high density of ohnologs with distinct set of CARs on either side of the red line.

**Fig S5.** Split of CAR 97. (A) Projections of CAR 97 on the human, spotted gar and chicken genomes, as well as its different assembly in the more recent version 84 of *Amniota*. These observations highlight the assembly error and shows that CAR 97 has to be split between gene 72 and gene 73, resulting in CAR 97a and CAR 97b. (B) The pairs of ohnologs that link CAR 97 to CAR 261 are drawn (dotted lines); three out of the five pairs involve ohnologs of CAR 97b and the two pairs left between CAR 97a and CAR 261 are in fact only one real pair because of a tandem duplication: this observation allows us to consider CAR 97a and CAR 261 as non ohnologous, therefore they were assembled, as all other conditions are fulfilled.

**Fig S6**. Split of CAR 46. The CAR 46 (77 genes) is drawn (in version 69) and the positions of the ohnologs in pair with CAR 123 are shown: they are all located in a small fragment that projects on human chromosome X and on LG1 of spotted gar, while the remaining part of the CAR maps on human chromosome 3 and on gar LG5, like CAR 123 with which CAR 46 is assembled on all other criteria but being ohnologous: this incited us to split CAR 46 in CAR 46a (gene 1 to 19) and CAR 46b (gene 20 to 77), the latter being assembled with CAR 123. Moreover the small CAR 46a fulfills all conditions to be assembled with CAR 66 at the same place in tetrad #7a (Figs. 12A and 12B).

**Fig S7**

See Fig S14 for legend.

**Fig S8**

See Fig S14 for legend.

**Fig S9**

See Fig S14 for legend.

**Fig S10**

See Fig S14 for legend.

**Fig S11**

See Fig S14 for legend.

**Fig S12**

See Fig S14 for legend.

**Fig S13**

See Fig S14 for legend.

**Fig S14**. **(A)** Representation of the tetrads before assembly of CARs**.** The numbers identify the CARs (arbitrarily numbered by AGORA) and the letter ‘a’ or ‘b’ characterizes the two segments after split of CARs 5, 118, 40, 97 and 46. The double arrows represent the ohnology links, solid lines indicating links that are supported by a Bonferroni-adjusted p-value of 0.05 (proportionality test). Dotted lines indicate links that are supported only without the Bonferroni correction, with three types of dotted lines used for three p-value thresholds. Numbers in black indicate CARs of at least 50 genes. Smaller CARs (< 50 genes) are in grey or in colors when several CARs are grouped together like in tetrad 7 in order to distinguish the links (to clarify which CARs are linked). **(B)** The same 17 tetrads after the 23 assemblies of the *Amniota* CARs (documented in Table 1, main text) that are part of the same ancestral chromosome. The assembled CARs are noted by “underscore” with arbitrary order between them. The legend is the same as in A for solid and dotted lines. The identifiers of the tetrads are indicated below each tetrad.

**Fig S15.** Diagram illustrating the mapping of the CLG human chromosome segments from Hg18 to Hg19 version of the human genome

([Putnam et al. 2008](#_ENREF_4)) constructed 17 chordate linkage groups that are considered as proto-chromosomes of the last common ancestor of amphioxus (*Branchiostoma floridae*) and vertebrates. Their content in human genes was deduced from the human chromosome segments that constitute them and for which the coordinates are available in version Hg18 of the human genome (([Putnam et al. 2008](#_ENREF_4)), Table S14). 120 human chromosome segments were incorporated into the CLGs, out of which 98 were directly converted by *liftOver*. The 22 remaining segments that are likely to be rearranged in Hg19 were divided each in 100 small segments that were subjected again to conversion by *liftOver*. Adjacent Hg19 segments were concatenated and their gene content recovered.

**Fig S16.** The 17 CLG are represented according to their intersection with the tetrads (pre-1R chromosomes). The size of the colored segments is proportional to the number of human genes of the CLG that are found in the corresponding tetrad (taking the human descendant of the ancestral genes assigned to the tetrad). Segments representing less than 10% of the CLG gene content were omitted except if they came from a tetrad that belonged to the same group as the predominant tetrad of the CLG. An exception was made for tetrad 13, that was not associated to a CLG: this tetrad contains only 203 human descendant genes out of which only 80 genes are found in a CLG; the 36 genes in CLG 5 (16% of the CLG genes) and the 30 genes in CLG 11 (3% of the CLG genes) are shown.

**Fig S17**. Genomicus display showing the pre-1R karyotype (top) with 17 chromosomes, the Amniote chromosome (middle) with 41 chromosomes larger than 45 genes, the chicken and the human karyotypes (bottom. Colors are defined according to human chromosomes, showing regions of homology in the other four genomes. Black lines in the ancestral Amniota and chicken chromosomes represent the conservation of gene order with human chromosomes. The pre-1R genes are not ordered. This display is entirely configurable, (more genomes can be added, etc) and can be accessed directly here: <https://tinyurl.com/y9jdtjga> while the Genomicus browser dedicated to this work can be accessed at this address : http://www.genomicus.biologie.ens.fr/genomicus-69.10/

**References.**

Braasch I, Gehrke AR, Smith JJ, Kawasaki K, Manousaki T, Pasquier J, Amores A, Desvignes T, Batzel P, Catchen J et al. 2016. The spotted gar genome illuminates vertebrate evolution and facilitates human-teleost comparisons. *Nature genetics* **48**(4): 427-437.

Makino T, McLysaght A. 2010. Ohnologs in the human genome are dosage balanced and frequently associated with disease. *Proceedings of the National Academy of Sciences* **107**(20): 9270-9274.

Nakatani Y, Takeda H, Kohara Y, Morishita S. 2007. Reconstruction of the vertebrate ancestral genome reveals dynamic genome reorganization in early vertebrates. *Genome Research* **17**(9): 1254-1265.

Putnam NH, Butts T, Ferrier DEK, Furlong RF, Hellsten U, Kawashima T, Robinson-Rechavi M, Shoguchi E, Terry A, Yu J-K et al. 2008. The amphioxus genome and the evolution of the chordate karyotype. *Nature* **453**(7198): 1064-1071.

Singh PP, Affeldt S, Cascone I, Selimoglu R, Camonis J, Isambert H. 2012. On the expansion of "dangerous" gene repertoires by whole-genome duplications in early vertebrates. *Cell reports* **2**(5): 1387-1398.

Smith JJ, Keinath MC. 2015. The sea lamprey meiotic map improves resolution of ancient vertebrate genome duplications. *Genome Res* **25**(8): 1081-1090.

Smith JJ, Timoshevskaya N, Ye C, Holt C, Keinath MC, Parker HJ, Cook ME, Hess JE, Narum SR, Lamanna F et al. 2018. The sea lamprey germline genome provides insights into programmed genome rearrangement and vertebrate evolution. *Nature genetics* **50**(2): 270-277.
